# Relaxation of the Ising spin system coupled to a bosonic bath and the time dependent mean field equation

**DOI:** 10.1371/journal.pone.0264412

**Published:** 2022-02-28

**Authors:** Máté Tibor Veszeli, Gábor Vattay

**Affiliations:** Institute of Physics, Eötvös University, Budapest, Hungary; Central State University & Ohio University, UNITED STATES

## Abstract

The Ising model does not have strictly defined dynamics, only a spectrum. There are different ways to equip it with time dependence, e.g., the Glauber or the Kawasaki dynamics, which are both stochastic, but it means there is a master equation that can also describe their dynamics. These equations can be derived from the Redfield equation, where the spin system is weakly coupled to a bosonic bath. In this paper, we investigate the temperature dependence of the relaxation time of a Glauber-type master equation, especially in the case of the fully connected, uniform Ising model. The finite-size effects were analyzed with a reduced master equation and the thermodynamic limit with a time-dependent mean field equation.

## 1 Introduction

Spin models are versatile because they are simple, yet able to demonstrate fundamental phenomena, like phase transition [1–3]. The spin models originated from solid state physics, where the interaction between the electron spins can be due to direct exchange [4], indirect exchange [5], superexchange [6] or double exchange [7], but many other complex physical systems can be modelled using a simple Ising or Heisenberg model, like nuclear spins [8, 9], and even social situations [10]. It is also important in modern applied physics since one branch of adiabatic quantum computers—e.g. the D-Wave system [11]—are based on finding the global minimum of an artificial spin system [12, 13].

The Ising model is defined via its energy or, in the quantum case, where it is called Heisenberg model via its Hamiltonian operator. All the equilibrium properties can be determined from these quantities, e.g. heat capacity, magnetic moment, susceptibility etc., since they can be derived from the partition function. On the other hand to calculate inequilibrium properties like the relaxation time, we need to know the dynamical equation of the system.

The Ising model does not have a natural dynamics, and although to the Heisenberg model we can associate the Schrödinger or the Heisenberg equation, it will only generate a unitary time evolution. Therefore it cannot be responsible for a final, thermal distribution. For this we need some interaction with the surrounding environment.

Close to thermal equilibrium it is often assumed, that the dynamics is driven by the gradient of a mean field free energy, since at equilibrium it must be zero [14–17]. This phenomenological approach can account for some dynamical critical behaviour, like the dynamical slowing down, but like every mean field approximation it is valid only if the fluctuations are small. For example in one dimension a master equation gives a better description [18].

A more fundamental approach is to derive the effective dynamical equations from a system-plus-bath model, where the bath is a thermal reservoir. This method let us track how the parameters of the macroscopic equation depends on microscopic properties. To describe such a system we must use the tools of open quantum systems [19, 20] like the Redfield [21] and the Lindblad equation [22]. These equations have countless applications in quantum biology [23, 24], quantum optics [19], cold atomic gases [25], chemical physics [26] as well as it also being relevant in quantum computing [27–29].

In solid matter the interaction with the phonons is always present and the electrons, as charged particles are coupled to the photons, thus we can assume that the spin system is in a bosonic bath. Quantum dissipation and relaxation of spin systems in a bosonic bath as well as in magnetic field have been investigated by many authors. [30–33].

In this paper the interaction between an adiabatic computer and its environment is meant to be small, so the weak coupling Lindblad equation is be used, but of course, there are improved methods to describe open quantum systems, like slippage initial condition [34, 35], the Nakajima-Zwanzig equation [36, 37] or the polaron transformation [38].

The structure of this paper is the following. In section 2 we briefly summarize the derivation of a Glauber-type master equation [39] based on the Redfield equation. Using this microscopic approach we can see how the Fourier transform of the bath correlation function appears in the final master equation. In section 3 we investigate the temperature dependence of the eigenvalues of the transition matrix because they contain relevant information on the time scales of the system, e.g., the relaxation time. We also give an upper bound to the smallest nonzero eigenvalue. In section 4 the dynamics of the uniform, fully connected Ising model is investigated, and we show that the relaxation time diverges in the thermodynamic limit as the temperature approaches the critical temperature. In section 5 a time dependent mean field equation is revisited, which is used in section 6 to extend the previous results to infinite sizes.

## 2 Master equation of quantum Ising system

In general if a system is connected to a bath, then its Hamiltonian operator is

$$\begin{array}{c} H_{\text{tot}}=H+H_{\text{B}}+H_{\text{I}}, \end{array}$$ (1)

where *H* acts only on the system of interest, *H*_B_ only on the bath, and *H*_I_ is the interaction between the two subsystems, it can be written as *H*_I_ = ∑_*α*_ *A*_*α*_ ⊗ *B*_*α*_, where *A*_*α*_ and *B*_*α*_ are system and bath operators respectively. The dynamics of the total system is described by the von Neumann equation.

$$\begin{array}{c} \rho_{\text{tot}}=-i\left[H_{\text{tot}},\rho_{\text{tot}}\right] \end{array}$$ (2)

After the Born and the Markov approximation an effective equation can be derived for the density operator of the system of interest. ($\rho ≔{\text{Tr}}_{\text{B}\rho_{\text{tot}}}$).

$$\begin{array}{c} \dot{\rho }\left(t\right)+i[H,\rho (t)]=\sum_{\alpha }(A_{\alpha }\rho \left(t\right)T_{\alpha }^{†}-A_{\alpha }T_{\alpha }\rho \left(t\right)+\text{h}.\text{c}.), \end{array}$$ (3)

where $T_{\alpha }=\sum_{\beta }\int_{0}^{\infty }dtC_{\alpha \beta }\left(t\right)A_{\beta }^{\text{I}}\left(-t\right)$, $A_{\beta }^{\text{I}}\left(t\right)$ is in interaction picture, $C_{\alpha \beta }\left(t\right)={\langle B_{\alpha }^{\text{I}}\left(t\right)B_{\beta }\rangle }_{\text{B}}$ is the bath correlation function, and h.c means hermitian conjugate. This is the Redfield equation in weak-coupling limit [21]. The Born approximation is valid if the interaction between the system and the bath is small, and we can use the Markov approximation if the relaxation time of the system of interest is much larger than the decaying time of the bath correlation function. After the so called secular or rotating wave approximation [40] one can get to the Lindblad equation [19, 22]:

$$\begin{array}{c} \dot{\rho }+i[H+H_{\text{LS}},\rho ]=\sum_{\alpha \beta }\sum_{\omega }\gamma_{\alpha \beta }\left(\omega \right)(A_{\beta }\left(\omega \right)\rho A_{\alpha }^{†}\left(\omega \right)-\frac{1}{2}\{A_{\alpha }^{†}\left(\omega \right)A_{\beta }\left(\omega \right),\rho \}), \end{array}$$ (4)

where $A_{\alpha }\left(\omega \right)=\sum_{ij}\left|i\rangle \langle i\right|A_{\alpha }\left|j\rangle \langle j\right|\delta_{\omega ,\varepsilon_{j}-\varepsilon_{i}}$ and |*i*〉 is the eigenvector of *H* with eigenvalue *ε*_*i*_. *H*_LS_ is the Lamb shift Hamiltonian, and *γ*_*αβ*_(*ω*) is the Fourier transform of the bath correlation function.

$$\begin{array}{c} \gamma_{\alpha \beta }\left(\omega \right)≔\int_{-\infty }^{\infty }\text{d}te^{i\omega t}{\left\langle B_{\alpha }^{†}\left(t\right)B_{\beta }\left(0\right)\right\rangle }_{\text{B}} \end{array}$$ (5)

**Fig 1:**
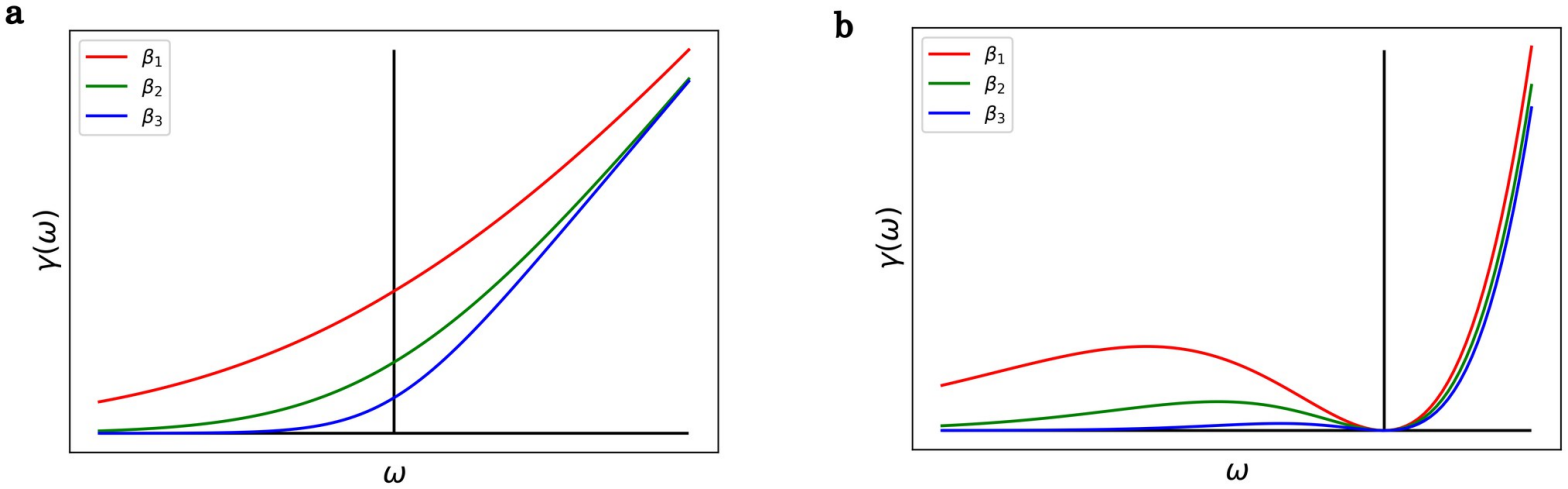
Fourier transform of the bath correlation function. *β*_1_ < *β*_2_ < *β*_3_. **a**) Ohmic bath. The *γ* function is monotonic, and *γ*(*ω* = 0) is finite. **b**) Super-Ohmic bath. The *γ* function is nonmonotonic, and *γ*(*ω* = 0) = 0.

There are two standard bosonic baths: the bath of phonons and the bath of photons. For phonons $\gamma_{\text{ohm}}\left(\omega \right)∼e^{-\frac{\left|\omega \right|}{\omega_{c}}}\frac{\omega }{1-e^{-\beta \omega }}$, which is called Ohmic case and for photons $\gamma_{\text{sup}}\left(\omega \right)∼\frac{\omega^{3}}{1-e^{-\beta \omega }}$, which is a super-Ohmic case. The frequency *ω*_*c*_ is the cutoff frequency. If we assume, that *ω*_*c*_ is large compared to the energy distances of the system, then $e^{-\frac{\omega }{|\omega_{\text{c}}|}}\approx 1$. Fig 1 shows the main features of the two *γ* functions. The main difference is that *γ*_ohm_ is strictly increasing and *γ*_ohm_(*ω* = 0) ∼ *k*_B_*T*, but *γ*_sup_ is non-monotonic, and *γ*_sup_(0) = 0. In the easiest case *γ*_*αβ*_ ∝ *δ*_*αβ*_.

The advantage of the weak-coupling limit is that a master equation can be derived for the diagonal elements of *ρ*.

$$\begin{array}{c} {\dot{P}}_{i}=\sum_{j}M_{ij}P_{j}\equiv \sum_{j}W_{ij}P_{j}-\sum_{j}W_{ji}P_{i}, \end{array}$$ (6)

where *P*_*i*_ = *ρ*_*ii*_, *W*_*ij*_ = ∑_*αβ*_ *γ*_*αβ*_(*ω*_*ji*_)(*A*_*α*_)_*ji*_(*A*_*β*_)_*ij*_ and *ω*_*ji*_ = (*ε*_*j*_ − *ε*_*i*_).

The system converges to the Boltzmann distribution if *W*_*ij*_ satisfies the detailed balance condition i.e., *W*_*ij*_ = *W*_*ji*_ exp(−*β*(*ε*_*i*_ − *ε*_*j*_)). Both the Ohmic and the super-Ohmic bath satisfy it, because

$$\begin{array}{c} \gamma \left(-\omega \right)={\text{e}}^{-\beta \omega }\gamma \left(\omega \right) \end{array}$$ (7)

If the system of interest is the Ising model, then a master equation can be derived [41], which contains only the diagonal elements of the density matrix. The Hamiltonian is

$$\begin{array}{c} H=-\sum_{\underset{\left(i<j\right)}{ij}}J_{ij}\sigma_{i}^{z}\sigma_{j}^{z}-\sum_{i}h_{i}\sigma_{i}^{z}, \end{array}$$ (8)

where $\sigma_{i}^{z}$ is the Pauli z-matrix and the corresponding eigenvectors are

$$\begin{array}{c} |\underline{S}\rangle \equiv |S_{1},S_{2},\ldots ,S_{N}\rangle \;S_{i}\in \left\{\pm 1\right\} \end{array}$$ (9)

with eigenenergies

$$\begin{array}{c} E_{\underline{S}}=-\sum_{\underset{\left(i<j\right)}{ij}}J_{ij}S_{i}S_{j}-\sum_{i}h_{i}S_{i} \end{array}$$ (10)

The easiest way to couple the system to the bath is via a Pauli matrix i.e., $A_{\alpha }\to \sigma_{i}^{x}$. Using *σ*^*z*^ in the interaction instead of *σ*^*x*^ would not give any relevant dynamics since the system and the interaction Hamiltonians would commute. The peculiarity of this system is that the populations decouple even without the secular approximation.

The $\sigma_{i}^{x}$ operator acting on |*S*〉 only flips the *i*th spin, so the *W*_*SS*′_ matrix element is

$$\begin{array}{c} \begin{array}{c} W_{\underline{S}\;{\underline{S}}^{\prime }}=\sum_{i}\gamma \left(\omega_{{\underline{S}}^{\prime }\underline{S}}\right){\left(\sigma_{i}^{x}\right)}_{{\underline{S}}^{\prime }\underline{S}}{\left(\sigma_{i}^{x}\right)}_{\underline{S}\;{\underline{S}}^{\prime }}=\{\begin{array}{ccc} \gamma (\omega_{{\underline{S}}^{\prime }\underline{S}}) & | & \;\text{if}\;\text{the}\;\text{Hamming}\;\text{distance}\;\\ & & \;\text{between}\;\underline{S}\;\text{and}\;{\underline{S}}^{\prime }\;\text{is}\;\text{1}\;\\ 0 & | & \text{otherwise.} \end{array} \end{array} \end{array}$$ (11)

With Eqs (6) and (11) we have a dynamics for the Ising model.

$$\begin{array}{c} {\dot{P}}_{\underline{S}}=\sum_{{\underline{S}}^{\prime }}M_{\underline{S}\;{\underline{S}}^{\prime }}P_{{\underline{S}}^{\prime }} \end{array},$$ (12)

where $M_{\underline{S}\;{\underline{S}}^{\prime }}=W_{\underline{S}\;{\underline{S}}^{\prime }}-\delta_{\underline{S}\;{\underline{S}}^{\prime }}\sum_{{\underline{S}}^{\prime \prime }}W_{{\underline{S}}^{\prime \prime }{\underline{S}}^{\prime }}$ is the transition matrix. This matrix is temperature dependent, and it has at least one zero eigenvalue, which is the eigenvalue of the equilibrium distribution:

$$\begin{array}{c} P_{\underline{S}}^{\text{eq}}=\frac{{\text{e}}^{-\beta E_{\underline{S}}}}{Z} \end{array}$$ (13)

For constant temperature the general solution of (12) is

$$\begin{array}{c} P_{\underline{S}}\left(t\right)=\sum_{{\underline{S}}^{\prime }}\sum_{\mu }{\text{e}}^{-\lambda_{\mu }t}P_{\mu ,\underline{S}}^{\text{R}}P_{\mu ,{\underline{S}}^{\prime }}^{\text{L}}P_{\underline{S}}\left(t=0\right), \end{array}$$ (14)

where $P_{\mu }^{\text{R}}\text{s}$ are the right, and $P_{\mu }^{\text{L}}\text{s}$ are the left eigenvectors of *M* with −λ_*μ*_ eigenvalues. All the λ_*μ*_s are nonnegative. If the system is ergodic, then there is only one zero eigenvalue, and the other λs are positive. Let the smallest positive be λ_min_ and the largest be λ_max_. The relaxation time is *t*_r_ = 1/λ_min_. This is the time scale in which all but the equilibrium mode dies out. The other relevant time scale is 1/λ_max_, which is the characteristic time of the fastest mode. If, for example, this spin system is a quantum computer, then the fastest mode is more important because if the computation is slower than this time scale, then the environment is not negligible. In other words, λ_min_ is important if we want the system to relax thermally, and λ_max_ is important if we want to avoid any thermal influence.

## 3 Temperature dependence of the eigenvalues

Both the smallest and the largest eigenvalues carry relevant information, and since *M*(*β*) is temperature dependent λ_min_(*β*) and λ_max_(*β*) are too.

At high temperatures we can determine the temperature dependence of all λs by simply Taylor expanding *γ*(*ω*; *β*) for small *β*.

$$\begin{array}{c} \gamma \left(\omega ;\beta \right)=\eta \frac{\omega^{\alpha }}{1-e^{-\beta \omega }}\approx \eta \frac{\omega^{\alpha -1}}{\beta }, \end{array}$$ (15)

where *α* = 1 in the Ohmic, and *α* = 3 in the super-Ohmic case. The transition matrix inherits this temperature dependence: *M*_*SS*′_(*β*) ∼ *β*^−1^, and hence λ ∼ *β*^−1^.

Despite the high-temperature limit, where the elements of the dynamical matrix *M* diverges, in the low-temperature limit they converge.

$$\begin{array}{c} \begin{array}{cc} \underset{\beta \to \infty }{\text{lim}}\gamma \left(\omega ;\beta \right)= & \left\{ \begin{array}{ccc} 0 & | & \omega \leq 0\\ \eta \omega^{\alpha } & | & \omega >0 \end{array}.\right. \end{array} \end{array}$$ (16)

It means all the eigenvalues also converge. As a consequence, we cannot slow down arbitrary all the modes by reducing the temperature. We have an upper limit in time for quantum computing. Of course, this calculation is valid only for a time-independent system, but the main features apply to more general cases.

Without an external magnetic field (*h* = 0) at zero temperature, the equilibrium Boltzmann distribution prefers only the two spin configurations with the lowest energies:

$$\begin{array}{c} P_{\underline{S}}^{\text{eq}}\left(T=0\right)=\frac{1}{2}\left(\delta_{\underline{S},{\underline{S}}_{\text{g}}}+\delta_{\underline{S},-{\underline{S}}_{\text{g}}}\right), \end{array}$$ (17)

where *S*_*g*_ and −*S*_*g*_ are the ground states. At zero temperature, there is one more eigenvector with a zero eigenvalue:

$$\begin{array}{c} P_{\text{min},\underline{S}}^{\text{R}}=\frac{1}{2}\left(\delta_{\underline{S},{\underline{S}}_{\text{g}}}-\delta_{\underline{S},-{\underline{S}}_{\text{g}}}\right) \end{array}$$ (18)

The question is how λ_min_(*β*) behaves at low temperature. We can give an upper bound. First let us introduce the following symmetric matrix:

$$\begin{array}{c} {\tilde{M}}_{\underline{S}\;{\underline{S}}^{\prime }}=M_{\underline{S}\;{\underline{S}}^{\prime }}\sqrt{\frac{P_{{\underline{S}}^{\prime }}^{\text{eq}}}{P_{\underline{S}}^{\text{eq}}}}\equiv M_{\underline{S}\;{\underline{S}}^{\prime }}e^{-\beta \frac{E_{{\underline{S}}^{\prime }}-E_{\underline{S}}}{2}} \end{array}$$ (19)

This transformation doesn’t affect the eigenvalues, and the eigenvectors transform like

$$\begin{array}{c} {\tilde{P}}_{\mu \underline{S}}=\frac{P_{\mu ,\underline{S}}^{\text{R}}}{\sqrt{P_{\underline{S}}^{\text{eq}}}}. \end{array}$$ (20)

Since $\tilde{M}$ is symmetric its right and left eigenvectors are the same, and now the variational method applies to it:

$$\begin{array}{c} \lambda_{\text{min}}\leq -\sum_{\underline{S},{\underline{S}}^{\prime }}{\tilde{\Pi }}_{\underline{S}}{\tilde{M}}_{\underline{S}\;{\underline{S}}^{\prime }}{\tilde{\Pi }}_{{\underline{S}}^{\prime }}, \end{array}$$ (21)

where $\tilde{\Pi }$ is an arbitrary vector with $\sum_{\underline{S}}{\tilde{\Pi }}_{\underline{S}}^{2}=1$, and it must be perpendicular to the equilibrium vector (${\tilde{P}}_{\underline{S}}^{\text{eq}}\equiv \sqrt{P_{\underline{S}}^{\text{eq}}}$), because λ_min_ is the second smallest eigenvalue of $\tilde{M}$. Let $\tilde{\Pi }=\frac{1}{\sqrt{2}}\left(\delta_{\underline{S}\;{\underline{S}}_{\text{g}}}-\delta_{\underline{S}\;-{\underline{S}}_{\text{g}}}\right)$. Then according to (21)

$$\begin{array}{c} \begin{array}{cc} \lambda_{\text{min}} & \leq -\frac{1}{2}\left({\tilde{M}}_{S_{\text{g}},S_{\text{g}}}-{\tilde{M}}_{S_{\text{g}},-S_{\text{g}}}-{\tilde{M}}_{-S_{\text{g}},S_{\text{g}}}+{\tilde{M}}_{-S_{\text{g}},-S_{\text{g}}}\right)\\ & =-{\tilde{M}}_{S_{\text{g}},S_{\text{g}}}=-M_{S_{\text{g}},S_{\text{g}}}=\sum_{S}W_{S,S_{\text{g}}}\\ & =\sum_{\underset{d(S,S_{\text{g}})=1}{S}}\gamma \left(\omega_{S_{\text{g}},S};\beta \right), \end{array} \end{array}$$ (22)

where *d*(*S*, *S*_g_) is the Hamming distance, and the *S* ↦ −*S* symmetry was used. In the bosonic bath

$$\begin{array}{c} \lambda_{\text{min}}\left(\beta \right)\leq \sum_{\underset{d(\underline{S},{\underline{S}}_{\text{g}})=1}{\underline{S}}}\eta {\left(\Delta E_{\underline{S}}\right)}^{\alpha }\frac{1}{{\text{e}}^{\beta \Delta E_{\underline{S}}}-1}, \end{array}$$ (23)

where $\Delta E_{\underline{S}}≔E_{\underline{S}}-E_{{\underline{S}}_{\text{g}}}>0$. At low temperatures this is the sum of some ${\text{e}}^{-\beta \Delta E_{\underline{S}}}$ functions, so λ_min_(*β*) can be estimated from above with an exponential function.

**Fig 2:**
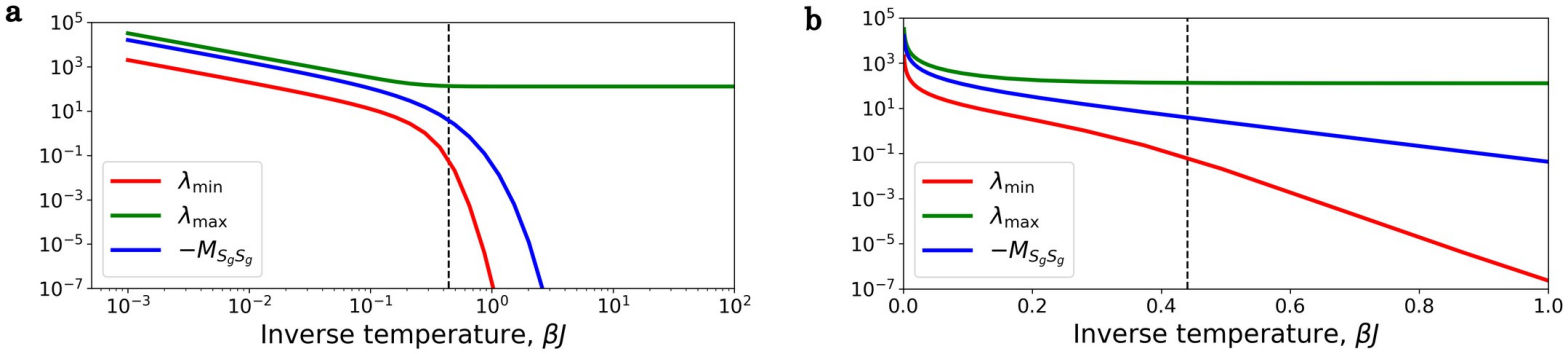
Temperature dependence of λ_min_, λ_max_ and $M_{{\underline{S}}_{\text{g}},{\underline{S}}_{\text{g}}}$. The system is the 2D, ferromagnetic, 4 × 4 Ising model, with Ohmic bath, where *J* = 1 and *η* = 1. **a**) log-log scale: At high-temperature all the eigenvalues have the λ ∝ *β*^−1^ temperature dependence. **b**) lin-log scale: At low-temperature λ_min_ decreases exponentially.

Fig 2 shows λ_min_(*β*), λ_max_(*β*) and $-M_{{\underline{S}}_{\text{g}},{\underline{S}}_{\text{g}}}$ (the upper bound) for a 4 × 4, ferromagnetic, 2D Ising model with Ohmic bath. The dashed vertical line marks the critical temperature ($\beta_{\text{c}}J=\frac{\text{ln}(1+\sqrt{2})}{2}\approx 0.44$). The left figure is in log-log scale, where we can see, that at low temperature the eigenvalues has a *β*^−1^ temperature dependence, and λ_max_(*β*) converges, and the right figure with lin-log scale shows, that λ_min_(*β*) goes to zero exponentially.

## 4 Eigenvalues of the uniform Ising model

The *M* matrices are 2^*N*^ × 2^*N*^ large; therefore, we cannot see how the eigenvalues behave at the thermodynamic limit. However, the uniform, fully connected Ising model is so symmetric that an effective equation can be derived with the same relaxation time as the original equation.

The energy of the model is

$$\begin{array}{c} E_{\underline{S}}=-\frac{J}{N}\sum_{\underset{\left(i>j\right)}{i,j=1}}^{N}S_{i}S_{j}. \end{array}$$ (24)

The 1/*N* factor is to keep the energy extensive and *J* > 0. Given an *S* microstate, it consists of *N*_↑_ spins with *S*_*i*_ = 1 and *N*_↓_ spins with *S*_*i*_ = −1. The number of spins is constant, i.e., *N*_↑_ + *N*_↓_ = *N* = fix. The energy of such a configuration is

$$\begin{array}{c} E_{\underline{S}}=-\frac{J}{N}[\frac{N_{↑}\left(N_{↑}-1\right)}{2}+\frac{N_{↓}\left(N_{↓}-1\right)}{2}-N_{↑}N_{↓}], \end{array}$$ (25)

If *N* is fixed, then the energy is the function of only *N*_↑_. The symmetry of the system is that we can permute the spins in any way, the energy and the *M* matrix remains the same. If in the dynamics the initial condition also has this symmetry, then the *P*_*S*_ will inherit this property. The slowest mode propagates between the two deepest valleys of the energy landscape, which are the ↑ ↑ …↑ and ↓↓ …↓. Assume that initially *P*_↓↓…↓_(*t* = 0) = 1, and we want to determine relaxation time, where *P*_↓↓…↓_(*t*_r_) ≈ *P*_↑ ↑…↑_(*t*_r_). Since both the equations and the initial condition have the permutation symmetry all the probabilities, which have the same up spin have the same value, e.g. for 3 spins *P*_↑↓↓_(*t*) = *P*_↓↑↓_(*t*) = *P*_↓↓↑_(*t*) ∀*t*. The probability can only flow between spin configurations if the Hamming distance between them is 1. Let us introduce the following probabilities:

PN↑=∑S_′PS_=(NN↑)P↑…↑︸N↑↓…↓︸N−N↑, (26)

where the prime denotes that only such configurations count where there are *N*_↑_ up spin. We can give a closed set of differential equations which only contain these new $P_{N_{↑}}$ probabilities.

$$\begin{array}{c} \begin{array}{cc} {\dot{P}}_{N_{↑}}= & \left(N-N_{↑}+1\right)W_{N_{↑},N_{↑}-1}P_{N_{↑}-1}\\ & +\left(N_{↑}+1\right)W_{N_{↑},N_{↑}+1}P_{N_{↑}+1}\\ & -(N_{↑}W_{N_{↑}-1,N_{↑}}+\left(N-N_{↑}\right)W_{N_{↑}+1,N_{↑}})P_{N_{↑}}, \end{array} \end{array}$$ (27)

where

$$\begin{array}{c} W_{N_{↑},N_{↑}+1}=\gamma \left(E_{N_{↑}+1}-E_{N_{↑}}\right)=\gamma (-\frac{2}{N}\left(2N_{↑}-N+1\right)). \end{array}$$ (28)

**Fig 3:**
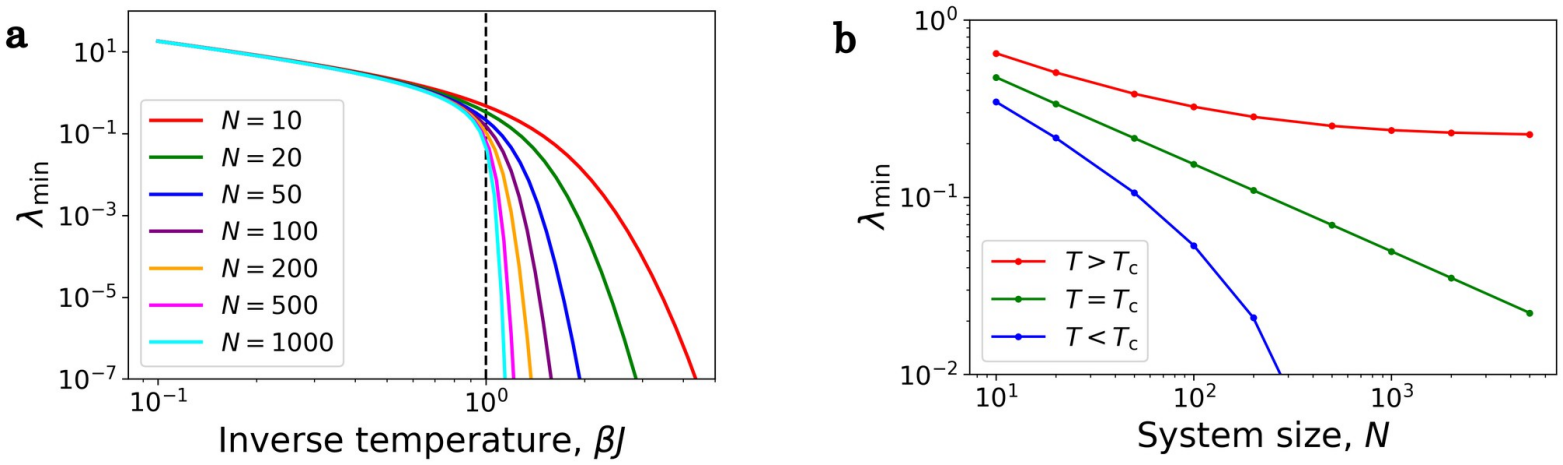
Smallest eigenvalue of *M*^red^ of the fully connected, uniform Ising model. The parameters of the system are *J* = 1 and *η* = 1. **a**) λ_min_ as a function of the inverse temperature for different system sizes. **b**) λ_min_ as a function of the system size for different temperatures. Above the critical temperature the curves converge, the relaxation time is finite in the thermodynamic limit. Below the critical temperature the relaxation time diverges.

This master equation has only *N* + 1 variables instead of 2^*N*^, thus is easy to simulate for large systems. A comparison between the quantum and the thermal simulated annealing of the fully connected Ising model was investigated by Wauters et al. using a similarly reduced master equation [42]. Eq (27) has the form

$$\begin{array}{c} {\dot{P}}_{N_{↑}}=\sum_{N_{↑}^{\prime }=0}^{N}M_{N_{↑},N_{↑}^{\prime }}^{\text{red}}P_{N_{↑}^{\prime }}, \end{array}$$ (29)

and we want to determine the lowest (nonzero) eigenvalue of *M*^red^, which is the same as the lowest (nonzero) eigenvalue of *M*. The matrix *M*^red^ is sparse, because it is a tridiagonal matrix, i.e., only the main diagonal, the first diagonal below and above the main diagonal are nonzero. Fig 3a shows the temperature dependence of λ_min_ for different system sizes. As *N* increases we can see, that around the critical temperature (which is *β*_c_*J* = 1) the behaviour of the system changes. At Fig 3b we can see better that above the critical temperature (*T* > *T*_c_) for large *N* values λ_min_ converges, meaning for every system size there is a finite relaxation time. At the critical temperature (*T* = *T*_c_), it follows a power law (λ_min_ ∝ *N*^−0.5^). Below the critical temperature (*T* < *T*_c_) λ_min_ goes to 0 for large *N*, but doesn’t follow a power law. This behaviour is the famous critical slowing down phenomenon.

**Fig 4:**
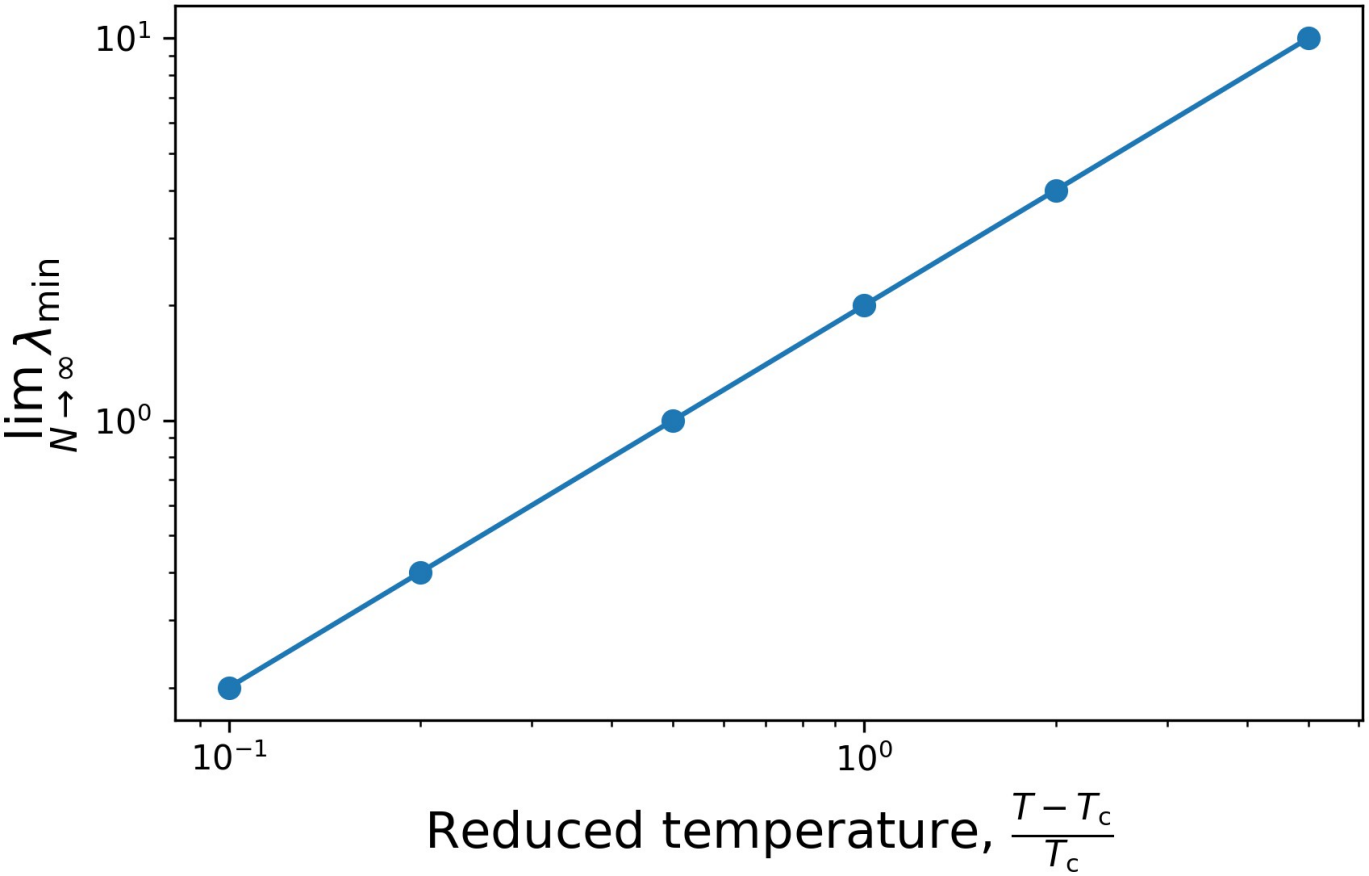
Critical behaviour of the fully connected Ising model above *T*_c_.

From the *N* → ∞ thermodynamic limit we can determine the dynamical critical exponent. Fig 4 shows λ_min_(*T*, *N* → ∞) as a function of the reduced temperature ($\frac{T-T_{\text{c}}}{T_{\text{c}}}$). This follows an easy power law, because λ_min_ ∝ *T* − *T*_c_. In the next section we will see that this result can be obtained from the mean field approximation.

## 5 Time dependent mean field equation

Since the primary interest is the magnetization (*m*_*i*_ ≔ 〈*S*_*i*_〉), we would like to derive a differential equation for it. Penrose showed that with some approximations, this is possible [43], and as the number of neighbors increases, this becomes more and more accurate.

Using the definition of *m*_*i*_ and the master equation we get

$$\begin{array}{c} \begin{array}{cc} {\dot{m}}_{i}= & \sum_{\underline{S}}{\dot{P}}_{\underline{S}}S_{i}=\sum_{\underline{S}\;{\underline{S}}^{\prime }}W_{\underline{S}\;{\underline{S}}^{\prime }}P_{{\underline{S}}^{\prime }}S_{i}-\sum_{\underline{S}\;{\underline{S}}^{\prime }}W_{{\underline{S}}^{\prime }\underline{S}}P_{\underline{S}}S_{i}^{\prime }\\ = & \sum_{{\underline{S}}^{\prime }\underline{S}}W_{{\underline{S}}^{\prime }\underline{S}}P_{\underline{S}}\left(S_{i}^{\prime }-S_{i}\right) \end{array} \end{array}$$ (30)

The *W*_*S*′*S*_ matrix component is nonzero if the Hamming distance between *S*′ and *S* is one. Introducing

$$\begin{array}{c} \begin{array}{c} \Lambda_{i}\left(\underline{S},n\right)=\{\begin{array}{ccc} S_{i} & | & i\neq n\\ -S_{i} & | & i=n \end{array} \end{array} \end{array}$$ (31)

we can rewrite the double sum in (30).

$$\begin{array}{c} \begin{array}{cc} {\dot{m}}_{i} & =\sum_{\underline{S}}\sum_{n=1}^{N}W_{\underline{\Lambda }\left(\underline{S},n\right),\underline{S}}\;P_{\underline{S}}\left(\Lambda_{i}\left(\underline{S},n\right)-S_{i}\right)\\ & =\sum_{\underline{S}}W_{\underline{\Lambda }\left(\underline{S},i\right),\underline{S}}\;P_{\underline{S}}\left(-2S_{i}\right)=-2\left\langle W_{\underline{\Lambda }\left(\underline{S},i\right),\underline{S}}S_{i}\right\rangle \end{array} \end{array}$$ (32)

In the second step the (Λ_*i*_(*S*, *n*) − *S*_*i*_) = 2*S*_*i*_ *δ*_*in*_ identity was used. The nonzero elements of *W* are a function of the energy difference:

$$\begin{array}{c} \begin{array}{cc} W_{\underline{\Lambda }\left(\underline{S},i\right),\underline{S}} & =\gamma (E_{\underline{S}}-E_{\underline{\Lambda }\left(\underline{S},i\right)})\\ & \equiv \gamma (-2{\tilde{h}}_{i}S_{i}), \end{array} \end{array}$$ (33)

where ${\tilde{h}}_{i}=\sum_{j}J_{ij}S_{j}+h_{i}$, so this is still the function of the *S* random variable, but because *J*_*ii*_ = 0 it is not a function of *S*_*i*_. Since *S*_*i*_ can be only 1 or −1 the $\gamma (-2{\tilde{h}}_{i}S_{i})$ as a function of *S*_*i*_ must have the

$$\begin{array}{c} \gamma \left(-2{\tilde{h}}_{i}S_{i}\right)\equiv \frac{\gamma \left(-2{\tilde{h}}_{i}\right)+\gamma \left(2{\tilde{h}}_{i}\right)}{2}+\frac{\gamma \left(-2{\tilde{h}}_{i}\right)-\gamma \left(2{\tilde{h}}_{i}\right)}{2}S_{i} \end{array}$$ (34)

form. Using (7) yields

$$\begin{array}{c} \begin{array}{cc} \gamma (-2{\tilde{h}}_{i}S_{i}) & =\gamma \left(2{\tilde{h}}_{i}\right)[\frac{{\text{e}}^{-2\beta {\tilde{h}}_{i}}+1}{2}+\frac{{\text{e}}^{-2\beta {\tilde{h}}_{i}}-1}{2}S_{i}]\\ & =\gamma \left(2{\tilde{h}}_{i}\right)\frac{{\text{e}}^{-2\beta {\tilde{h}}_{i}}+1}{2}[1-\text{tanh}\left(\beta {\tilde{h}}_{i}\right)S_{i}], \end{array} \end{array}$$ (35)

then substituting back to (32) gives

$$\begin{array}{c} {\dot{m}}_{i}=-\langle \gamma \left(2{\tilde{h}}_{i}\right)({\text{e}}^{-2\beta {\tilde{h}}_{i}}+1)(S_{i}-\text{tanh}\left(\beta {\tilde{h}}_{i}\right))\rangle . \end{array}$$ (36)

Eq (36) is similar to the Callen equation [44, 45]$\left(\langle S_{i}\rangle =\langle \text{tanh}\left(\beta {\tilde{h}}_{i}\right)\rangle \right)$, where the averaging is outside the hyperbolic function. In order to get a closed equation for the expected values, the average must move inside, and instead of the *S*_*i*_ random variables, their *m*_*i*_ expected values must be written.

$$\begin{array}{c} \begin{array}{c} {\dot{m}}_{i}=-\gamma (2(\Sigma_{j}J_{ij}m_{j}+h_{i}))(1+{\text{e}}^{-2\beta (\sum_{j}J_{ij}m_{j}+h_{i})})\\ \times (m_{i}-\text{tanh}(\beta (\Sigma_{j}J_{ij}m_{j}+h_{i}))) \end{array} \end{array}$$ (37)

This mean field approximation is valid as long as the fluctuations are small, which holds if every spin interacts with many other spins. This can be due to long range interaction or in high spatial dimensions [46]. The right-hand side of Eq (37) contains the self-consistent equation from the equilibrium statistical physics; hence if the equation of state is satisfied, then ${\dot{m}}_{i}=0$.

Eq (37) contains both the real-time and the temperature of the bath. The temperature can also be time-dependent, and in that case, we could get thermal annealing, but if the temperature is constant, we can determine the relaxation time and the dynamical critical exponent. If *m*(*t*) = *m*^eq^ + *δm*(*t*), where *m*^eq^ is the equilibrium solution and *δm*(*t*) is small, then the linearized equation of (37) is

$$\begin{array}{c} \begin{array}{c} \delta {\dot{m}}_{i}=-b_{i}\left({\underline{m}}^{\text{eq}}\right)\sum_{j}\{(\delta_{ij}-\frac{\beta J_{ij}}{{\text{cosh}}^{2}\left(\beta \sum_{k}J_{ik}m_{k}^{\text{eq}}+h_{i}\right)})\delta m_{j}\}, \end{array} \end{array}$$ (38)

where

$$\begin{array}{c} b_{i}\left({\underline{m}}^{\text{eq}}\right)=\gamma (2(\Sigma_{j}J_{ij}m_{j}^{\text{eq}}+h_{i}))(1+{\text{e}}^{-2\beta (\sum_{j}J_{ij}m_{j}^{\text{eq}}+h_{i})}). \end{array}$$ (39)

Using the $\frac{1}{{\text{cosh}}^{2}\left(x\right)}\equiv 1-{\text{tanh}}^{2}\left(x\right)$ identity, and the equation of state we get to

$$\begin{array}{c} \delta {\dot{m}}_{i}=-b_{i}\left({\underline{m}}^{\text{eq}}\right)\sum_{j}\{(\delta_{ij}-\beta J_{ij}(1-{\left(m_{j}^{\text{eq}}\right)}^{2}))\delta m_{j}\}. \end{array}$$ (40)

Eq (40) contains the inverse susceptibility of the mean field Ising model.

$$\begin{array}{c} \chi_{ij}^{-1}≔\frac{\partial^{2}F^{\text{MFA}}}{\partial m_{i}\partial m_{j}}=-J_{ij}+\frac{T\delta_{ij}}{1-m_{i}^{2}}, \end{array}$$ (41)

where

$$\begin{array}{c} \begin{array}{cc} & F^{\text{MFA}}\left(\underline{m},\underline{h},T\right)=-\frac{1}{2}\sum_{ij}J_{ij}m_{i}m_{j}-\sum_{i}h_{i}m_{j}\\ & +T\sum_{i}[\frac{1+m_{i}}{2}\text{ln}(\frac{1+m_{i}}{2})+\frac{1-m_{i}}{2}\text{ln}(\frac{1-m_{i}}{2})]. \end{array} \end{array}$$ (42)

Substituting the inverse susceptibility back into (40) yields

$$\begin{array}{c} \begin{array}{c} \delta {\dot{m}}_{i}=-b_{i}\left({\underline{m}}^{\text{eq}}\right)\beta (1-{\left(m_{i}^{\text{eq}}\right)}^{2})\sum_{j}\chi_{ij}^{-1}\delta m_{j}\equiv -\Gamma_{i}\sum_{j}\chi_{ij}^{-1}\delta m_{j}. \end{array} \end{array}$$ (43)

This is a well-known equation in the theory of dynamical critical phenomena [47], but it is usually derived from the $\dot{\underline{m}}=-\Gamma \partial_{\underline{m}}F^{\text{MFA}}$ phenomenological equation. Now we can see, how it is related to a master equation and the spin-boson model. If the system is symmetric in a sense, that all the spins behave the same, then (43) simplifies to

$$\begin{array}{c} \delta \dot{m}=-\Gamma \chi^{-1}\delta m, \end{array}$$ (44)

where $\chi^{-1}=\sum_{j}\chi_{ij}^{-1}$.

## 6 Time dependent mean field equation for the uniform Ising model

As before in section 4 the uniform Ising model will be studied because in the equilibrium case in the thermodynamic limit, it gives back the exact results. According to (42) the mean field free energy is

$$\begin{array}{c} \begin{array}{c} \frac{F^{\text{MFA}}\left(m,h,T\right)}{N}=-\frac{1}{2}Jm^{2}-hm+T(\frac{1+m}{2}\text{ln}(\frac{1+m}{2})+\frac{1-m}{2}\text{ln}(\frac{1-m}{2})), \end{array} \end{array}$$ (45)

and the time dependent mean field equation is

$$\begin{array}{c} \begin{array}{c} \dot{m}=-\gamma \left(2\left(Jm+h\right)\right)(1+{\text{e}}^{-2\beta (Jm+h)})\times (m-\text{tanh}(\beta (Jm+h))). \end{array} \end{array}$$ (46)

If *h* = 0 the critical temperature is *T*_c_ = *J*, and above this temperature the equilibrium solution is *m*^eq^ = 0. The inverse susceptibility is

$$\begin{array}{c} \chi^{-1}=-J+T\equiv T-T_{\text{c}}, \end{array}$$ (47)

therefore

$$\begin{array}{c} \lambda_{\text{min}}=\Gamma \left(T-T_{\text{c}}\right), \end{array}$$ (48)

where Γ = 2*γ*_ohm_(0; *β*)*β* = 2*η* in the Ohmic bath. In the super-Ohmic bath, this is zero because *γ*_sup_(0) = 0, i.e., there is no transition between states with the same energy. In a real physical system it means that besides the Glauber dynamics some other effects are not negligible, e.g. 2 spin flips. Eq (48) is the same result that we have already seen in Fig 4. As in equilibrium statistical physics, the mean field approximation gives back the exact result for the uniform model in the thermodynamic limit.

At the critical temperature the inverse susceptibility is zero, the linear term vanishes, and we need the higher order terms. Taylor expanding (46) at *T* = *T*_c_ around *m* = *m*^eq^ ≡ 0 up to the third order gives.

$$\begin{array}{c} \delta \dot{m}=-\frac{2}{3}\eta J\delta m^{3} \end{array}$$ (49)

which has the

$$\begin{array}{c} \delta m\left(t\right)\propto t^{-\frac{1}{2}} \end{array}$$ (50)

solution for large *t*s, which means there isn’t a characteristic time.

Below the critical temperature, the linearized equation is good again; only *m*^eq^ changes. On the other hand Eq (46) can give a different solution from the (29) master equation. If we want to compare these two equations, the initial condition must also be the same, which gives a restriction to the initial condition of (29). In the mean field approximation, the probability is a product of the one-particle probabilities:

$$\begin{array}{c} P_{\underline{S}}^{\text{MFA}}=\prod_{i}\frac{1+m_{i}S_{i}}{2}, \end{array}$$ (51)

which means the initial probability of (29) is

PN↑(t=0;m)=(NN↑)(1+m2)N↑(1−m2)N↓ (52)

**Fig 5:**
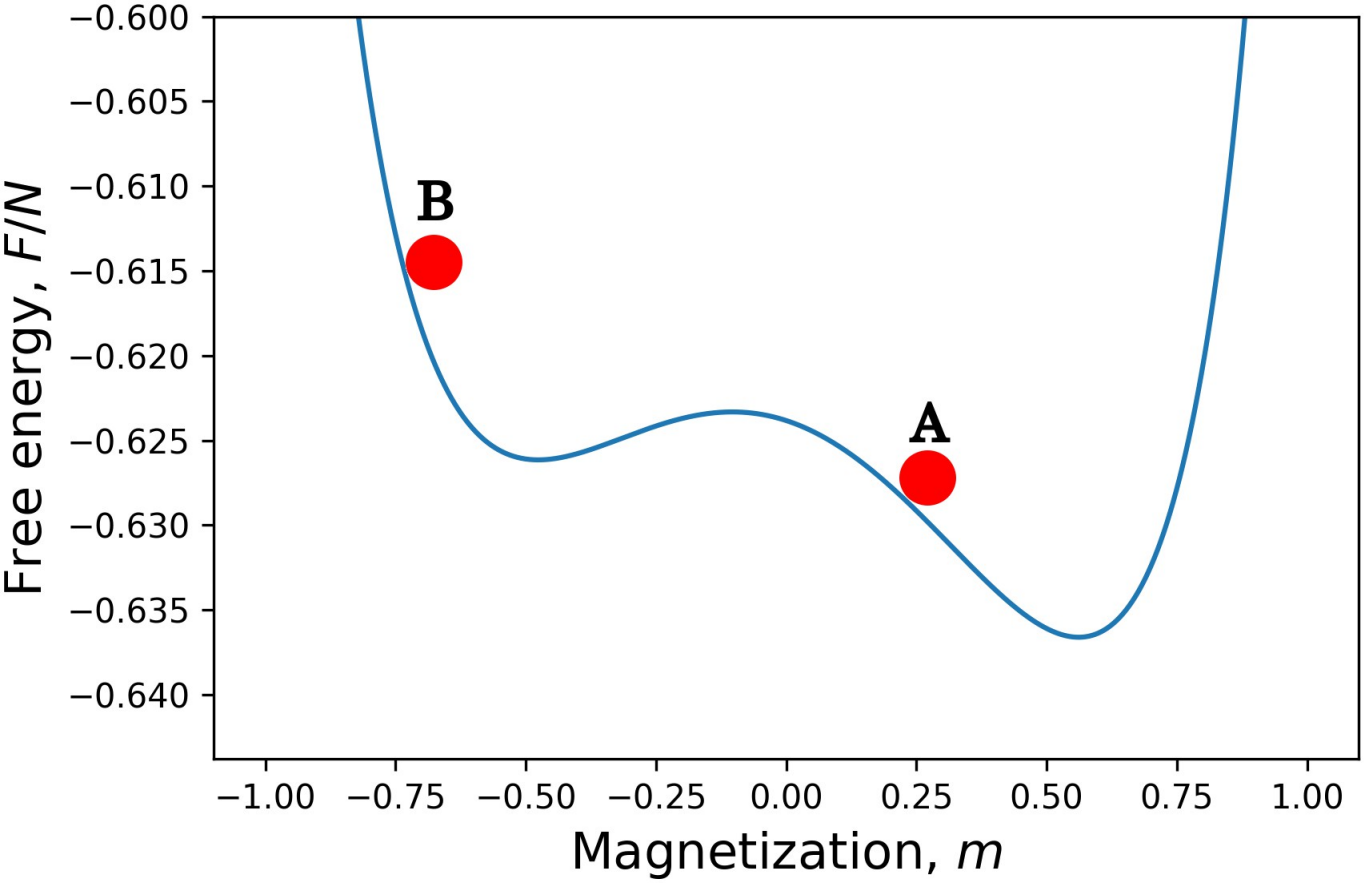
Free energy of the uniform Ising model with finite external magnetic field.

**Fig 6:**
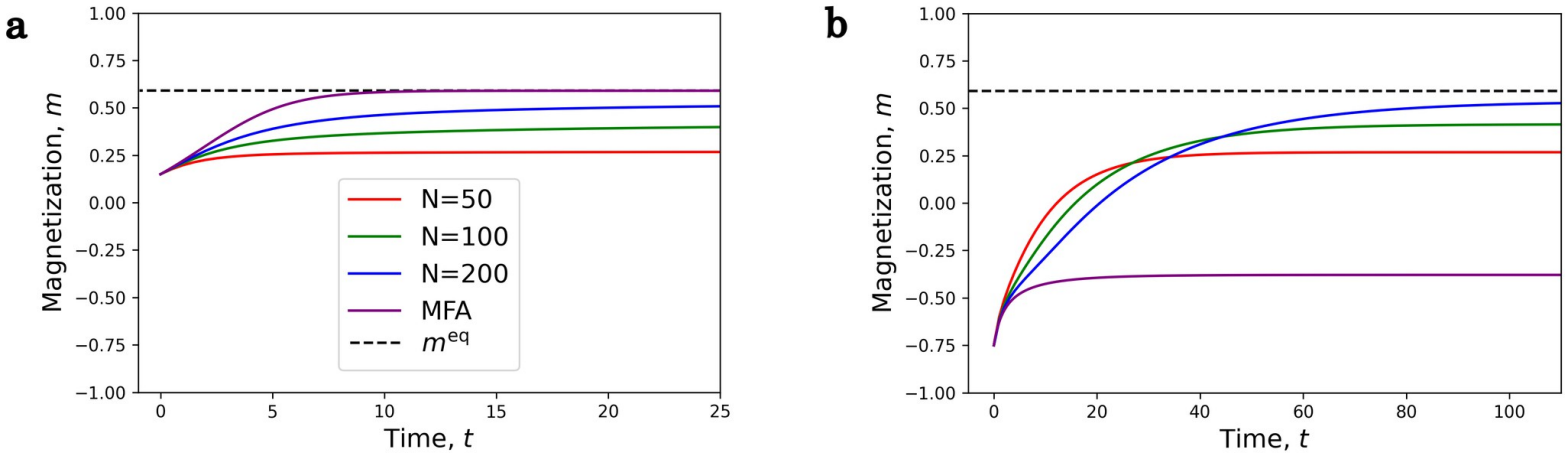
Comparison of the time dependent mean field equation and the master equation. The parameters are *J* = 1, *η* = 1, *h* = 0.02, *T* = 0.9, and Ohmic bath was used.**a**) Point A, If the initial *m* is close to the global minimum, then for large as *N* increases the solution of the master equation converges to the solution of the mean field equation.**b**) Point B, If the initial *m* is close to the local minimum, then the relaxation time diverges for large *N* values for the master equation, and the mean field equation converges to the local minimum.

If *h* = 0, then the master equation must converge to the *m* = 0 solution, but the time-dependent mean field equation finds one of the minima of the free energy if initially *m* ≠ 0. If *h* is finite and *m* is in the valley of the global minimum (point A in Fig 5), then in the *N* → ∞ limit the solution of the master equation converges to the mean field solution (Fig 6).

On the other hand, if initially, *m* is in the valley of the local minimum (point B in Fig 5), then the solution of the mean field equation converges to this local minimum, but the solutions of the master equation are different. The probabilities converge to the equilibrium

PN↑eq(h,N)∝(NN↑)e−βEN↑(h,N) (53)

distribution, so they tend to approach *m*^eq^ for large *N* values in the *t* → ∞ limit, but as *N* grows, so does the relaxation time. In the thermodynamic limit the relaxation time diverges as in Fig 3. In the *N* → ∞ limit the solution of the master equation converges to the solution of the mean field equation and none of them will approach the global minimum, because the relaxation time will be infinite.

## 7 Conclusion

In this work, we have studied a Glauber-type master equation derived from the spin-boson model. The most relevant dynamical properties are encoded in the eigenvalues of the transition matrix of the master equation. They are temperature dependent and behave significantly differently below, above, and at the critical temperature as a function of the system size. In the case of the uniform, fully connected Ising model, in the thermodynamic limit, above the critical temperature the relaxation time follows a power law: *t_r_* ∝ (*T* − *T_c_*)^−1^.

Using the time-dependent mean field equation, we could also investigate the thermodynamic limit. Its dynamics differ significantly from the finite size master equation if the initial condition is close to a local minimum of the free energy, which means that the relaxation time diverges. If it is close to the global minimum, then the solution of the finite size master equation converges to the solution of the time-dependent mean field equation.
